# Massachusetts Medical College

**Published:** 1864-04

**Authors:** 


Massachusetts Medical College.—At the commencement held on the 9th of March, 1864, the degree of M. D. was conferred on 38 candidates.

